# Display of *Bombyx mori* Alcohol Dehydrogenases on the *Bacillus subtilis* Spore Surface to Enhance Enzymatic Activity under Adverse Conditions

**DOI:** 10.1371/journal.pone.0021454

**Published:** 2011-06-29

**Authors:** Nan Wang, Cheng Chang, Qin Yao, Guohui Li, Lvgao Qin, Liang Chen, Keping Chen

**Affiliations:** Institute of Life Sciences, Jiangsu University, Zhenjiang, Jiangsu Province, People's Republic of China; University of Connecticut, United States of America

## Abstract

Alcohol dehydrogenases (ADHs) are oxidoreductases catalyzing the reversible oxidation of alcohols to corresponding aldehydes or ketones accompanied by nicotinamide adenine dinucleotide (NAD) or nicotinamide adenine dinucleotide phosphate (NADP) as coenzyme. ADHs attract major scientific and industrial interest for the evolutionary perspectives, afforded by their wide occurrence in nature, and for their use in industrial synthesis. However, the low activity of ADHs under extremes of pH and temperature often limits their application. To obtain ADH with high activity, in this study, we used *Bombyx mori* alcohol dehydrogenases (*BmADH*) as foreign gene and constructed a recombinant integrative plasmid pJS700-*BmADH*. This pJS700-*BmADH* was transformed into *Bacillus subtilis* by double cross-over and produced an amylase inactivated mutant. The fusion protein containing BmADH was expressed on the spore surface and recognized by BmADH-specific antibody. We also assayed the alcohol dehydrogenase activity of the fusion protein together with the native BmADH at different pH and temperature levels, which indicated the recombinant enzyme exhibits activity over wider ranges of temperature and pH than its native form, perhaps due to the resistance properties of *B. subtilis* spores against adverse conditions.

## Introduction

Alcohol dehydrogenases (ADH; EC 1.1.1.1) belong to the oxidoreductase family; a class of enzymes that catalyze the reversible oxidation of alcohols to corresponding aldehydes or ketones using NAD or NADP as coenzyme. ADHs are widely distributed in nature and have been found in species throughout the three domains of life, Archaea, Bacteria and Eukarya [1]-[4]. ADHs play important roles in a broad range of physiological process. Based on their catalytic activities, ADHs are supposed to participate in the metabolism of steroids, retinoids, lipid peroxidation products, ω-hydroxy fatty acids, xenobiotic alcohols and aldehydes [5]. However, many ADHs are generally susceptive to harsh conditions such as extremes of pH and temperature, which often hampers their industrial application [6]. Due to the ever-increasing industrial demands,microbial surface display of enzymes has been widely adopted as a promising technique for the improvement of conventional biocatalysts [7]-[9].

The gram-positive bacterium *Bacillus subtilis* has been extensively studied as a model prokaryotic system, which is not regarded as a pathogen but classified as a novel food currently being used as a probiotic for both human and animal consumption [10], [11]. The *B. subtilis* spore offers unique resistance properties and can survive extremes of temperature, desiccation, exposure to solvents and other noxious chemicals. These attributes would make the spore an attractive vehicle for delivery of heterologous proteins that are resistant to adverse environments. Additionally, three proteins in the spore coat, CotB, CotC and CotG, have been shown to be exposed on the outside and have been used as a novel system to display passenger proteins such as different antigens [12], enzymes [13] and bioactive molecules [14].

In this study, a mutant *B. subtilis* strain, which exhibited *Bombyx mori* alcohol dehydrogenase (BmADH) on the spore surface by fusion to the spore coat protein CotC, has been successfully constructed, expressed and identified by a BmADH-specific antibody. The ethanol dehydrogenase activity of the BmADH protein expressed on the spore surface was assayed under different pHs and temperatures and this enzyme's activity was compared with that of the native enzyme under the same conditions.

## Methods

### Materials

*B. subtilis* 168 (trp-) was obtained from Bacillus Genetic Stock Center, Department of Biochemistry, The Ohio State University. The expression vector pET-30a(+) and *Escherichia coli* strains DH5α and BL21(DE3) were obtained from Novagen (CA, USA). Ex Taq polymerase, restriction enzymes, T4 DNA ligase and the subcloning vector pMD18-T were purchased from TaKaRa (Dalian, China). Chemicals are all from Sigma (MO, USA) or a domestic provider in China if not stated otherwise.

Preparation and transformation of *B. subtilis* strain 168 (trp-) competent cells were performed as previously described [15].

### Expression of BmADH in *E. coli* and preparation of the specific antibody

The BmADH specific primers, *BmADH*-F1: 5′-GGGGATCCATGGCACCGGATTTCGTG-3′ (*Bam* HI site is underlined), and *BmADH*-R1: 5′-CCCTCGAGCTATGTCTTGGAGAGTATTTGGAAG-3′ (*Xho* I site is underlined) were designed to amplify an 825 bp *BmADH* gene from a pool of silkworm cDNA. The PCR product was ligated into the pMD18-T vector and then subcloned into the pET-30a(+) expression vector and transformed into *E*. *coli* BL21 (DE3). *E. coli* cells containing pET30a-*BmADH* were grown to an optical density at about 0.6 at 600 nm and induced by addition of 0.4 mM IPTG at 16°C for 20 hours to obtain soluble protein, and cells were harvested by centrifugation at 8,000 g for 15 min at 4°C. The soluble his-tagged protein was purified by a Ni-NTA column (Qiagen, German) following manufacturer's instruction. The recombinant BmADH present in the pellet of cell lysate was mixed with SDS sample buffer, boiled for 5 min and resolved on 15% SDS-polyacrylamide gels. The recombinant BmADH was excised and extracted from gel slices. The identity of the BmADH protein was confirmed by mass spectroscopic analysis and the purity was examined by a SDS-PAGE. The antiserum was raised in New Zealand white rabbits according to the method of Sambrook et al. using Freund's adjuvant [16]. All the procedures were in line with ethical standards for treatment of animals.

### Construction of plasmid pJS700-*BmADH*

To obtain an integration of the *CotC-BmADH* fusion gene at the *B. subtilis amyE*, a recombinant plasmid for double cross-over with *B. subtilis* chromosome was constructed. We amplified another 825 bp fragment from silkworm cDNA with primer pair *BmADH*-F2: 5′-GGGGTACCATGGCACCGGATTTCGTGAAGCGTT-3′ (*Kpn* I site is underlined), and *BmADH*-R2: 5′-CGAGCTCCTATGTCTTGGAGAGTATTTGGA-3′ (*Sac* I site is underlined). The fragment between the *Kpn* I and *Sac* I sites containing the *BmADH* gene was cloned into vector pJS700, to generate the recombinant plasmid pJS700-*BmADH*, in which the upstream and downstream region of *erythromycin (Em)-CotC-BmADH* was homologous to *B. subtilis amyE*, as was verified by sequencing [17].

Plasmid pJS700-*BmADH* was digested with *Bgl* II, and the resulted linear fragment containing *Em-CotC-BmADH* gene was transformed into the *B. subtilis* strain 168 (trp-) competent cells. The transformed cells were incubated in 2 ml LB medium and cultured at 37°C overnight with vigorous shaking, following with sprayed onto LB plate containing 0.4 mg/ml erythromycin. Plates were incubated at 37°C overnight, colonies resistant to Em were selected, and a colony with *CotC-BmADH* integrated at the *B. subtilis amyE* locus was identified by analysis of amylase activity and then confirmed by PCR.

### Screening of mutants with *CotC-BmADH* integrated at *amyE* locus

The integration of *CotC-BmADH* at *amyE* locus will disrupt *amyE* gene causing amylase-negative phenotype on LB plates containing 1% starch [15]. After incubation at 37°C overnight, the plates were stained by iodine to examine the amylase activity. A blue color was produced by starch-iodine reaction in the presence of *CotC–BmADH* fusion in the *B. subtilis* chromosome. However, no blue color was observed at the *B. subtilis* 168 (trp-) control when iodine was added, since the expression of *amyE* resulted in hydrolysis of the starch in the plate.

Chromosomal DNA was made from both of the *B. subtilis* 168 (trp-) control and *CotC-BmADH* transfromants for site-directed PCR. Primers *amyE*-F: 5′-GAGATCTCATTGCTCGGGCTGTATGACTGG-3′ and *amyE*-R: 5′-GTTACACCATCACTGTTCGTTCC-3′ will amplify a 1098 bp wild-type fragment and a 4.5 kb integrated fragment. Primer pair *BmADH*-F2 and *BmADH*-R2 was used to detect the correct insertion of the *BmADH* gene.

### Expression of CotC-BmADH on *B. subtilis* spore surface

Sporulation of wild type *B. subtilis* 168 (trp-) and recombinant strain CotC-BmADH was induced in Difco sporulation medium (DSM) by the exhaustion method as previously described [18]. Cultures were harvested 48 h after the initiation of sporulation. Spores were collected, washed several times, and purified by lysosome treatment as described by Nicholson and Setlow to break any residual sporulating cells [18]. Spore coat proteins in suspensions at high density (>1×10^10^ spores/ml) were extracted from *B. subtilis* 168 (trp-) spores and the CotC-BmADH spores using an SDS–DTT extraction buffer as previously described [15].

### SDS-PAGE and western blotting

Extracted coat proteins were mixed with SDS-PAGE loading buffer and run on a 12% polyacrylamide gel at room temperature. Electrophoresis was at 70 V for 30 min and then at 120 V for 120 min. The gel was stained with 0.25% Coomassie blue R-250 for 3 h and then destained for 6 h using a mixture of 10% acetic acid and 5% ethanol. To confirm that BmADH was expressed on the *B. subtilis* spore surface, the proteins in the gel were transferred to a polyvinylidene fluoride (PVDF) membrane (Millipore) for western blotting using previously prepared BmADH-specific antibody at a dilution of 1:1000. Immunoreactive proteins were visualized using goat anti-rabbit IgG antibody conjugated with peroxidase as described [19].

### Enzymatic activity assay

The catalytic activity of recombinant CotC-BmADH protein was assayed spectrophotometrically by measuring the increase in absorbance at 340 nm following the reduction of NAD^+^ to NADH in a solution containing ethanol as the substrate as described by Oudman *et al.* [20]. Briefly, a 3 ml reaction buffer (50 mM NaOH/Glycine buffer, pH 10.0, 0.67 M ethanol, 8 mM NAD^+^) was incubated at 25°C, and the reaction was initiated by adding 0.1 ml of spore suspensions. The supernatant of the reaction solution was clarified by centrifugation (12,000 g, 4°C, 30 s) for spectrophotmetric analysis. The increase at A_340_ in the initial 10 minutes was recorded. The increase in A_340_ for reactions without spores was used as the blank. An extinction coefficient of 6.22 mM^-1^ cm^-1^ for NADH was used to calculate enzyme activity [21]. One unit of dehydrogenase activity is defined as 1 µmol NAD^+^ reduced per min. The enzyme specific activity calculation formula is as following: (△A_340_×V) / (6.22×b×W),where △A_340_ is the change in absorbance at 340 nm per minute ,V is the final reaction volume, b is the light path, and W is the amount of protein in the reaction system. The concentration of extracted proteins was measured using the Bio-Rad DC Protein Assay kit. Spores of *B. subtilis* 168 (trp-) were used as negative control. The enzymatic activity was measured three times with independent samples.

Alcohol dehydrogenase specific activity at different pH levels was determined by performing standard enzyme assay at various pH levels ranging from 4.0 to 10.0. The following 0.1 M buffer systems of varying pH, but fixed ionic strength, were used: acetate buffer (NaAc-HAc) for pH 4.0; phosphate buffer (NaH_2_PO_4_–Na_2_HPO_4_) for pH 7.0; NaOH/Glycine buffer for pH 8.0, 9.0 and 10.0.

Alcohol dehydrogenase specific activity at different temperature was determined by performing standard enzyme assays for 10 min at temperatures of 16, 20, 25, 30, and 37°C.

To verify the thermal stability of the native BmADH and spore-displayed CotC-BmADH, the two proteins were incubated respectively at 37°C for 30 min, and then assayed under optimal conditions. The relative remained activity was calculated by comparison with the activity of the untreated proteins.

### Statistical analysis

The enzymatic activity difference between the native BmADH and CotC-BmADH was analyzed by t-test with a significance level of *α* = 0.05 (Microsoft Excell in Office 97). All values are expressed as mean value ± standard deviation of three independent experiments.

## Results

### Construction of integrative vector containing *CotC-BmADH* gene

**Figure 1:**
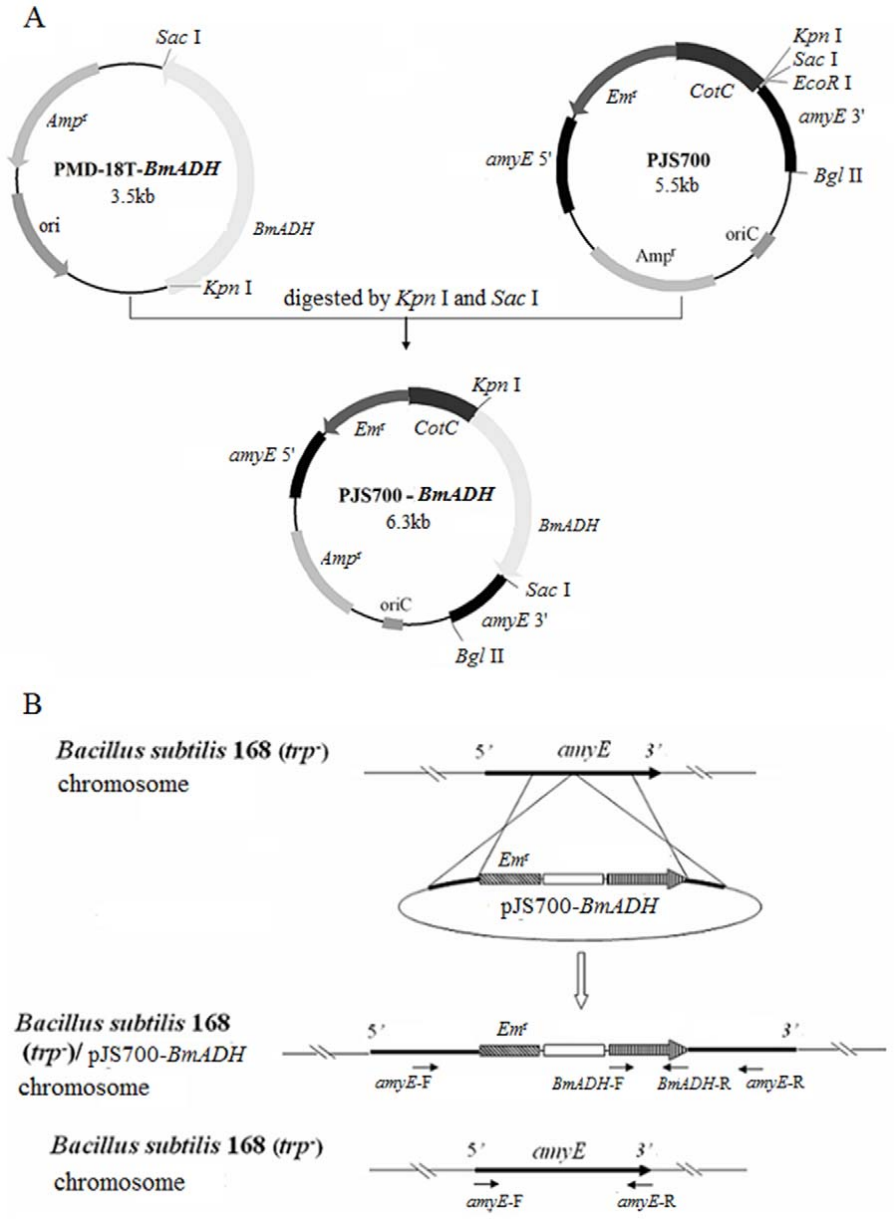
Cloning strategy. (A) The construction of integration plasmid pJS700-BmADH. The fragments *amyE* 5′ and *amyE* 3′ in plasmid are homologous to the upstream and downstream of the amylase gene in *B. subtilis* 168 (trp-), respectively; *Em* ^r^, erythromycin resistant site; *CotC*, a *B. subtilis* spore coat protein encoding gene. (B) The schematic integration of CotC-BmADH to *amyE* locus. Arrows indicate the positions of primer pairs used in the site-directed PCR for confirmation of the correct integration.

The strategy to obtain recombinant *B. subtilis* spores expressing BmADH on their surface was based on (i) use of the *CotC* gene and its promoter for the construction of translational fusions and on (ii) chromosomal integration of the *CotC-BmADH* gene fusions into the coding sequence of the *amyE* gene [15]. The *CotC-BmADH* gene was obtained by cloning the *BmADH* gene at the 3′ end of the 201-bp-*CotC* open reading frame carried by the integrative vector pJS700 (Fig. 1A). The fusion was integrated into the *B. subtilis* chromosome at the *amyE* locus by a double cross-over event (Fig. 1B).

### Identification of *CotC-BmADH* integrated in *B. subtilis* chromosome

**Figure 2:**
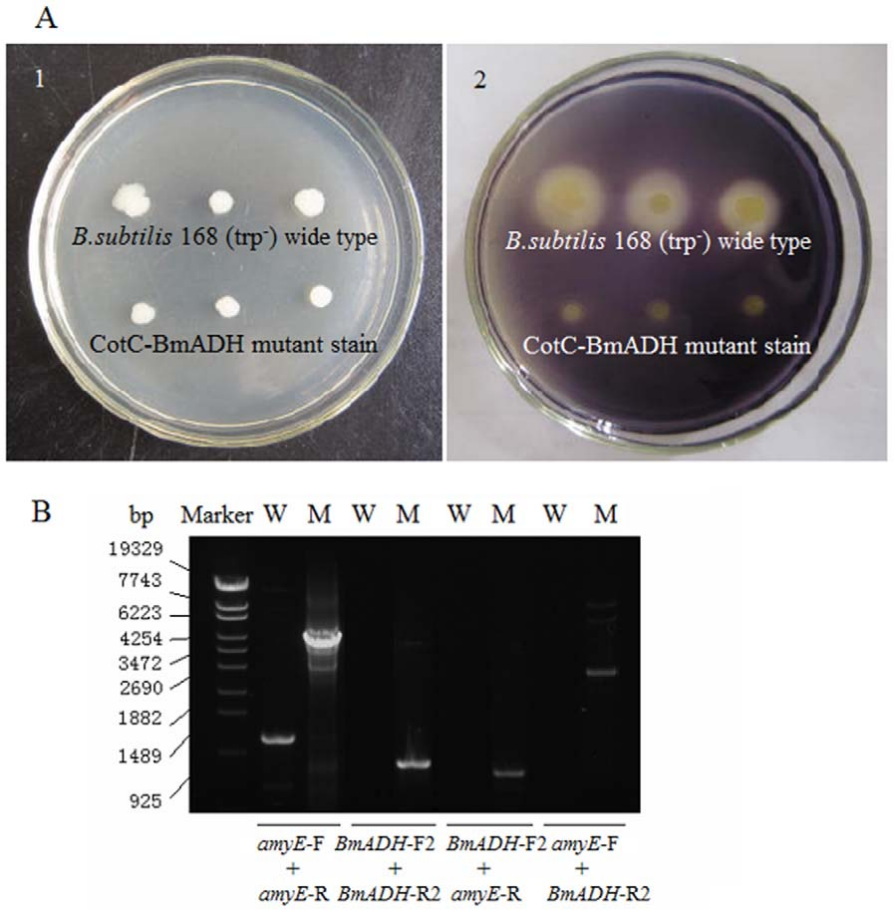
Identification of the mutant with CotC-BmADH integration at *amyE* locus. (A) Analysis of amylase activity. CotC-BmADH mutant strains and *B. subtilis* 168 (trp-) wide type grew on the starch-containing LB plate before (1) and after (2) being stained by iodine. The integration of CotC-BmADH might disrupt *amyE* and made the strain amylase deficient, while the while wide type strain showed a big white halo around colony due the secretion of amylase. (B) Site-directed PCR analysis using different primer pairs. Marker, *λ* DNA digested by *Eco*T14I; W: *B. subtilis* 168 (trp-) wide typ; M: CotC-BmADH mutant; primer pairs used in PCR are labeled below agarose gel.

Amylase activity assay was used to identify *CotC-BmADH* integration mutants. The integration of *CotC-BmADH* at *amyE* locus disrupted the production of amylase, as a result no white halo was observed around the colony on a starch-containing plate stained by iodine, but the control strain showed a big white halo around the colony due to the secretion of amylase (Fig. 2A). The disruption of *amyE* was further confirmed by PCR with different primer pairs (Fig. 2B). Primer pair *amyE*-F/*amyE*-R produced a 4.5 kb fragment from the *CotC-BmADH* integrated chromosome, in comparison with a 1098 bp fragment from the *B. subtilis* 168 (trp-) DNA. In addition, in the *CotC-BmADH* integrated chromosome, primer pairs *BmADH*-F/*BmADH*-R, *BmADH*-F/*amyE*-R, *amyE*-F/*BmADH*-R produced fragments around 825 bp, 700 bp, 2650 bp, respectively, but gave no PCR product with 168 (trp-) DNA.

### Surface display of BmADH on the recombinant spores

**Figure 3:**
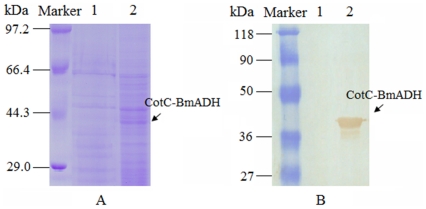
SDS-PAGE analysis of CotC-BmADH and Western blotting. (A) SDS-PAGE stained by coomassie-blue. (B) CotC-BmADH detected by BmADH specific antibody. Lane 1, *B. subtilis* 168 (trp-); lane 2, CotC-BmADH strain.

To confirm that BmADH was expressed on the *B. subtilis* spore surface, approximate 5 µg coat proteins were loaded to perform SDS-PAGE analysis, which showed a distinctive protein band weights about 40 kDa (Fig. 3A). Because the *CotC* gene of *B. subtilis* encodes a 66 amino acid polypeptide (CotC) with a predicted molecular mass of 8.8 kDa [22], and the 825 bp ORF of BmADH encodes a 275 amino acid protein with a predicted molecular mass of 30 kDa, the SDS-PAGE analysis suggested that the recombinant fusion protein was expressed with expected molecular mass.

The expression of CotC-BmADH was confirmed by Western blotting with previously prepared polyclonal antibody. A 40 kDa band was detected in the extracts from recombinant spores, while no similar band in the control lane was detected, indicating the presence of the CotC–BmADH fusion protein on the spore surface (Fig. 3B).

### Effect of pH and temperature on enzyme activity

**Figure 4:**
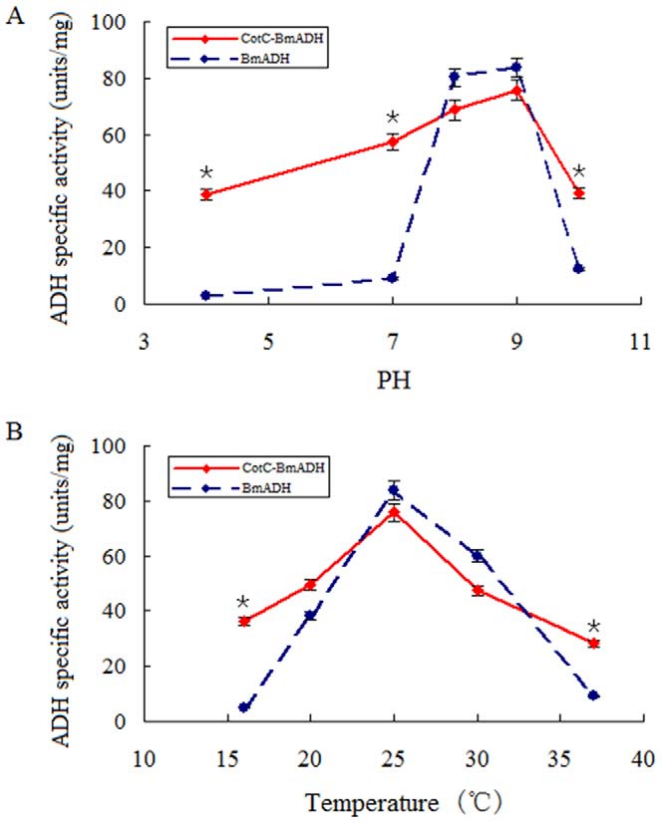
Alcohol dehydrogenase specific activity at different pHs (A) and temperatures (B). Solid line indicates CotC-BmADH; dotted line indicates the native BmADH. Mean ± SD from three independent experiments are shown. *indicates the difference is significant (P<0.05).

By measuring the alcohol dehydrogenase specific activity of both CotC-BmADH and the native BmADH under standard enzyme assay conditions at various pH levels, we found that the spore-displayed enzyme exhibited significant higher specific activity at pH 4.0, 7.0 and 10.0 than that of the native enzyme (P<0.05), which indicated that the spore-displayed enzyme retained activity in a wider range of pH than the native protein (Fig. 4A, Table 1).

**Table 1:** Alcohol dehydrogenase specific activities at different pHs.

protein samples	pH				
	4	7	8	9	10
BmADH (units/mg protein)	3.05±0.35	9.14±0.82	80.22±4.59	83.77±3.27	12.23±1.37
CotC-BmADH (units/mg protein)	38.64±1.14*	57.58±2.38*	68.72±3.29	75.82±0.82	39.36±3.82*

*indicates that the difference is significant (P<0.05).

Mean ± SD from three independent experiments are shown.

By measuring the alcohol dehydrogenase specific activity of both CotC-BmADH and the native BmADH under standard enzyme assay conditions at various temperatures, we found that the spore-displayed enzyme exhibited significant higher specific activity at 16°C and 37°C than that of the native enzyme (P<0.05), which indicated that the CotC-BmADH preserved activity in a wider range of temperature than the native protein (Fig. 4B, Table 2).

**Table 2:** Alcohol dehydrogenase specific activities at different temperatures.

protein samples	Temperature (°C)				
	16	20	25	30	37
BmADH (units/mg protein)	5.08±0.08	38.07±1.57	83.77±5.83	60.00±3.30	9.14±1.60
CotC-BmADH (units/mg protein)	36.35±1.15*	49.60±2.37	75.82±5.42	47.32±1.78	28.24±1.16*

*indicates that the difference is significant (P<0.05).

Mean ± SD from three independent experiments are shown.

**Figure 5:**
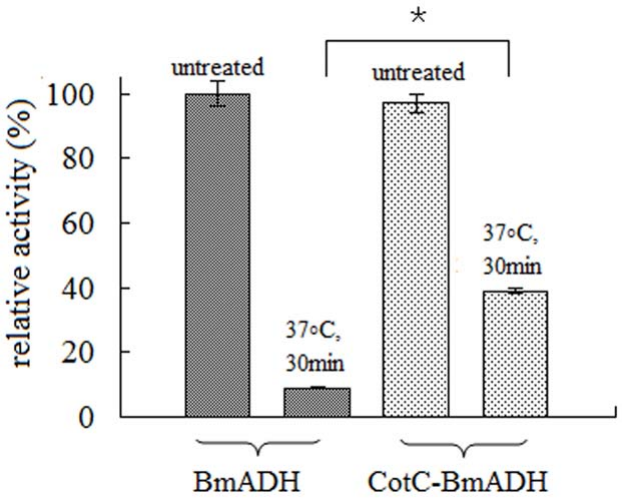
Relative remained activity of BmADH and CotC-BmADH after incubation at 37°C for 30 min. The activity was assayed at pH 9.0, 25°C. *indicates the difference is significant (P<0.05).

In addition, the protein stability of CotC-BmADH was compared with that of the native BmADH at 37°C. The catalytic activities of CotC-BmADH and BmADH were measured at optimal condition after incubation of protein at 37°C for 30 min. The measurement revealed that the spore-displayed protein retained approximate 40% of its original activity, while 10% activity was remained for the native BmADH (Fig.5), which suggesting that the fusion of BmADH to *B. subtilis* spore protected the protein from the irreversible destructive conformational change at 37°C.

## Discussion

The *Bacillus subtilis* spore is encased within a complex multilayered protein structure known as the coat, whose role is to protect the spore against bactericidal enzymes and chemicals, and to influence the spore's ability to germinate in response to appropriate germinants. The coat is composed of more than 20 polypeptides, several of which have been studied, and their structural genes (*cot* genes) have been identified [23], [24]. The development of a spore surface display system was initially based on the use of CotB, a *B. subtilis* spore coat component, as a fusion partner to express a highly immunogenic tetanus toxin fragment C (TTFC) on the spore surface [25]. More recently, another coat component, CotC, was also tested as fusion partner for the expression of TTFC and of the B subunit of the heatlabile toxin of *E. coli* (LTB) [26], [27]. In this study, we displayed BmADH on the *B. subtilis* Spore surface by fusing BmADH to the C-end of CotC protein, and the fusion protein exhibited catalytic activity over wider ranges of pH and temperature than its native form. This improvement may be contributed by the resistant properties of *B. subtilis* spore, which provided protection to the bioactive molecules against harsh conditions.

The alcohol dehydrogenase activity assay revealed that the highest activities of the modified BmADH and the native BmADH were at pH 8.0–9.0. It is worth mentioning that this result coincides with the data in the literature regarding the optimum pH for alcohol dehydrogenase [28], [29]. In addition, the optimum temperature of 25°C for BmADHs was consistent with the body temperature of silkworm. Moreover, CotC-BmADH was able to catalyze in extremes of pH and temperature, while the activity of BmADH decreased dramatically out of the optimal range. However, we noticed that the maximum specific activity of the fusion protein was a little lower than that of the native protein. This might be caused by the larger molecular mass of CotC-BmADH, as well as other proteins in the spore.

In conclusion, this study has provided a detailed picture of the molecular strategy that allows environmental susceptive enzymes to survive adverse conditions by forming robust *B. subtilis* spores. The results showed our method could yield enzyme with higher stability and activity than its native form, and suggested this method could be expanded to the industrial application of enzymes.
